# Coherent anti-Stokes Raman scattering imaging of lipids in cancer metastasis

**DOI:** 10.1186/1471-2407-9-42

**Published:** 2009-01-30

**Authors:** Thuc T Le, Terry B Huff, Ji-Xin Cheng

**Affiliations:** 1 Weldon School of Biomedical Engineering, Purdue University, West Lafayette, IN 47907, USA; 2 Department of Chemistry, Purdue University, West Lafayette, IN 47907, USA; 3 Purdue Cancer Center, Purdue University, West Lafayette, IN 47907, USA

## Abstract

**Background:**

Lipid-rich tumours have been associated with increased cancer metastasis and aggressive clinical behaviours. Nonetheless, pathologists cannot classify lipid-rich tumours as a clinically distinctive form of carcinoma due to a lack of mechanistic understanding on the roles of lipids in cancer development.

**Methods:**

Coherent anti-Stokes Raman scattering (CARS) microscopy is employed to study cancer cell behaviours in excess lipid environments *in vivo *and *in vitro*. The impacts of a high fat diet on cancer development are evaluated in a Balb/c mice cancer model. Intravital flow cytometry and histology are employed to enumerate cancer cell escape to the bloodstream and metastasis to lung tissues, respectively. Cancer cell motility and tissue invasion capability are also evaluated in excess lipid environments.

**Results:**

CARS imaging reveals intracellular lipid accumulation is induced by excess free fatty acids (FFAs). Excess FFAs incorporation onto cancer cell membrane induces membrane phase separation, reduces cell-cell contact, increases surface adhesion, and promotes tissue invasion. Increased plasma FFAs level and visceral adiposity are associated with early rise in circulating tumour cells and increased lung metastasis. Furthermore, CARS imaging reveals FFAs-induced lipid accumulation in primary, circulating, and metastasized cancer cells.

**Conclusion:**

Lipid-rich tumours are linked to cancer metastasis through FFAs-induced physical perturbations on cancer cell membrane. Most importantly, the revelation of lipid-rich circulating tumour cells suggests possible development of CARS intravital flow cytometry for label-free detection of early-stage cancer metastasis.

## Background

Excess lipid in the body has been shown to aggravate cancer. Animal studies showed that high fat diets and obesity enhanced cancer metastasis [1]. In human, a body mass index above 30 kg/m^2 ^is strongly correlated with increased risk for various types of cancer [2]. It is generally accepted that diet and obesity are accountable for 30% of preventable causes of cancer [2]. Indeed, diet, nutrition, and physical activity are widely promoted as effective means for cancer prevention by the World Cancer Research Fund and the American Institute for Cancer Research [3]. Nonetheless, the relationship between diet and cancer incidence and mortality in human remains controversial due to conflicting experimental results from clinical studies [4,5]. Currently, a consensus is lacking on the benefits of a certain type of fatty acid or nutritional ingredient to cancer prevention [6]. A contributing factor to the controversy is the lack of mechanistic understanding on how excess lipid or adiposity affects cancer development [2,6].

Excess lipid at the cellular level has also been associated with cancer aggressiveness. Intracellular lipid body accumulation has been observed in many types of cancers including mammary, brain, adrenal, and others [7-9]. Early clinical studies in the 1970s of patients with lipid-rich carcinoma of mammary glands found high incidence of cancer mortality, metastatic tumours, and other aggressive clinical behaviours [7]. Since then, lipid-rich carcinoma continued to be reported widely in human and animal [10,11]. Nonetheless, the relationship between intracellular lipid accumulation and cancer behaviour has not been investigated. Without a mechanistic understanding of the role of lipid in cancer development, pathologists are refrained from classifying lipid-rich tumours as a morphologically and clinically distinctive form of carcinoma [10].

To investigate the roles of lipid in cancer development, we employ a lipid-sensitive imaging technique called coherent anti-Stokes Raman scattering (CARS) microscopy [12]. CARS is a four wave mixing process where two synchronized lasers, pump and Stokes, are tightly focused into a diffraction-limited focal volume. The interaction of the pump field at frequency ω_p _and the Stokes field at frequency ω_S _with the medium generates an anti-Stokes field at frequency 2ω_p_-ω_S_. CARS signal is significantly enhanced when ω_p_-ω_S _matches a Raman-active vibrational band. Furthermore, the intrinsic coherent property allows CARS signal to increase quadratically with respect to the number of molecular vibrations in the focal volume. Such property renders CARS highly sensitive to lipid-rich structures when ω_p_-ω_S _matches the symmetric CH_2 _stretch vibration at 2840 cm^-1^. CARS microscopy has been widely applied as a label-free imaging technique to visualize lipid bilayers, cell membranes, adipocytes, myelin sheaths, foam cells of atheroma, and others [13,14]. An additional unique advantage of CARS microscopy is its intrinsic capability for multimodal imaging. A typical CARS microscope with picosecond pulse excitation is capable of simultaneous CARS, sum frequency generation (SFG), and two-photon excitation fluorescence (TPEF) imaging. Such multimodal imaging capability has allowed characterization of the impact of obesity on the composition and architecture of mammary tumour stroma [15]. Here, CARS microscopy is employed to elucidate the mechanistic link between lipid-rich tumours and aggressive tumour behaviours.

## Methods

### Multimodal nonlinear optical microscopy

A multimodal nonlinear optical (NLO) microscope capable of CARS, SFG, and TPEF imaging on a single platform has been previously described [15]. For CARS imaging, the wave number difference ω_p_-ω_S _was tuned to 2840 cm^-1 ^which matches the Raman shift of symmetric CH_2 _stretch vibration. The same CARS laser sources were used for SFG and TPEF imaging. CARS, SFG, and TPEF (green fluorescence/DIOC18/FITC/GFP) signals were collected through a 600/65 nm (Ealing Catalog, Rocklin, CA, Cat. No. 42-7336), a 375/50 nm (Chroma, Rockingham, Vermont, Cat. No. HQ375/50), and a 520/40 nm (Chroma, Rockingham, Vermont, Cat. No. HQ520/40) bandpass filters, respectively. To image TPEF signal for red fluorescence (Rh-DOPE or RFP), CARS laser sources were desynchronized such that there was no contribution from CARS signal to fluorescence signal. Red fluorescence TPEF signal was collected through a 600/65 nm (Ealing Catalog, Rocklin, CA, Cat. No. 42-7336) bandpass filter. For SFG and TPEF imaging, backward-reflected signals were collected. For CARS imaging of cell cultures, forward signals (F-CARS) were collected. For CARS imaging of tissue samples, backward-reflected signals (E-CARS) were collected. The combined laser power at the sample was kept constantly at 40 mW.

### Tumour cell line and growth medium

Madison (M109) lung carcinoma cell line of Balb/c mice origin was a generous gift from P. Low (Purdue Cancer Center, Purdue University, West Lafayette, IN). M109 cells were grown in RPMI-1640 medium supplemented with 10% fetal bovine serum and antibiotics penicillin (50 U)/streptomycin (50 μg). A dual-color labelled M109 cell line was created by stable transfection of M109 cells with a dual-reporter plasmid which expresses both GFP and RFP.

### Animal model

To evaluate the impact of diet on tumour metastasis, Balb/c mice (6–8 weeks old) were subcutaneously injected with M109 cells (1 million cells in 0.1 ml PBS buffer per mouse) in the hind leg area. Then mice (week 0) were placed on two types of diet, a lean diet (20 mice) and a high fat diet (12 mice). Lean diet (Harlan Teklad, Indianapolis, IN, Cat. No. 7001) has 4.25% fat and 3.82 Kcal/g. High fat diet (Research Diets, New Brunswick, New Jersey, Cat. No. D12492) has 34.9% fat and 5.24 Kcal/g. On week 2, 4 mice from each diet group were sacrificed and visceral fat weights were measured. For the remaining mice, weight, tumour size, and number of circulating tumour cells were monitored for 4 weeks after tumour implantation. After 4 weeks, 8 mice from each diet group had terminal blood samples drawn to analyze for circulating tumour cells and fatty acid levels. Lung and primary tumour tissues were also collected and analyzed with nonlinear optical imaging or with histology. 8 mice on normal diet were kept until week 5, then lung tissues were collected for lung metastasis analysis. Furthermore, 4 mice on normal diet and 4 mice on high fat diet were injected with sterilized PBS buffer (0.1 ml) in the hind leg area for control experiments. All animal experiments were approved by Purdue Animal Care and Use Committee.

### A dual-reporter plasmid to label M109 cells

A dual-reporter plasmid was constructed to label M109 cells with both GFP and RFP. A ~3.5 kb DNA fragment is generated by total synthesis (Genscript, Piscataway, NJ) and cloned into a pDSRed-Express-DR plasmid (Cat. No. 632423, Clontech, Mountain View, CA) between restriction sites XhoI and BamHI. This DNA fragment comprises a 1.4 kb fragment of vascular endothelial growth factor (VEGF) promoter (nucleotides -963 to +404) controlling the expression of a GFP (0.8 kb, Cat. No. 632428, Clontech, Mountain View, CA), a SV40 polyA transcription termination signal (0.25 kb), and a 1 kb fragment of collagen type I (col1a1) promoter (nucleotides -915 to +116). In the final plasmid construct, GFP expression is controlled by VEGF promoter and RFP expression is controlled by col1a1 promoter. The excitation and emission maxima for GFP are 496 nm and 506 nm, and for RFP are 557 nm and 579 nm, respectively.

### Stable transfection of M109 cells

M109 cells at density of 1 × 10^5 ^in 35 mm culture dishes were transfected with dual-reporter plasmid DNA using Fugene6 transfection reagent (Cat. No. 11815091001, Roche Diagnostics, Indianapolis, IN) at the ratio of 6 μl of Fugene6 reagent to 1 μg DNA. At 24 hours after transfection, cells were selected with 400 ng/ml Geneticin G418 Sulfate (Cat. No. 10131, Invitrogen, Carlsbad, CA) until individual colonies were observed under a microscope. Colonies were detached from culture dishes by incubation with non-enzymatic cell dissociation reagent (Cat. No. 1676949, MP Biomedicals, Solon, OH) for 5 minutes at 37°C. Individual colonies were carefully removed using pipette tips under a microscope. To ensure a single clone is used, a selected colony was diluted into multi-well plates at the concentration of one cell per well and re-grown in 100 ng/ml Geneticin G418 Sulfate (Cat. No. 10131, Invitrogen, Carlsbad, CA).

### Intravital flow cytometry

To enumerate the number of circulating tumour cells in the blood, intravital flow cytometry in superficial veins of a mouse ear was employed. Using F-CARS or transmission microscopy, a vein of ~8 μm in diameter which has a steady flow rate of ~1000 cells/min was selected. This small diameter vein ensures that blood cells of ~5 μm in diameter flow through the vein in a single file. Additionally, fluctuation in detection signal due to varying cell position along the vertical axis of the detection volume is minimized because of restricted vessel volume. A laser scanning line (~8 μm) which spans the entire vein diameter was defined perpendicular to the direction of blood flow. The laser scanning speed of ~1.3 ms/line ensured sampling of all flow-through blood cells. In each mouse, two different veins were selected for measurement. Four sampling windows of ~1 minute per window were used to enumerate circulating tumour cells in each mouse. The average count of circulating tumour cells and the standard deviation between sampling windows were plotted for each mouse against time after tumour implantation. To visualize dual-labeled circulating tumour cells, a 488 nm Argon laser was used to excite GFP and a 543 nm Helium-Neon laser was used to excite RFP simultaneously. Emission from GFP and RFP were collected through a 520/40 nm and 600/65 nm filters, respectively. The power of each laser was kept constantly at 100 μW at the sample. Images were acquired using Fluoview software and processed using ImageJ software. High frequency noise was removed using ImageJ despeckle and Gaussian filter functions. The detection accuracy of circulating tumour cells is enhanced by the cross-correlation of signals arising from both GFP and RFP within the same cell.

### Isolation and identification of circulating tumour cells

At 4 weeks after tumour implantation, mice were subjected to terminal blood collection. On average, approximately 1 ml of blood was collected from each mouse. Whole blood was centrifuged at 300 × g for 10 minutes at 4°C to separate plasma from blood cells. After plasma was removed for FFAs quantitation and GC-MS analysis, pellets were reconstituted in 10 ml red blood cell lysis buffer (eBioscience, San Diego, CA, Cat. No. 00-4333-57) and incubated for 5 minutes at room temperature. Then 20 ml of PBS buffer was added to stop lysis reaction. The sample was centrifuged at 300 × g for 10 minutes at 4°C to remove lysed red blood cells from other blood components. Pellets were reconstituted in supplemented RPMI medium and plated onto a glass-bottom chamber for NLO imaging. To detect circulating tumour cells (CTC), simultaneous F-CARS imaging for lipid detection and epi-reflected TPEF imaging for green fluorescent protein (GFP) detection were performed. Cells screened positive for lipid and GFP signals were further imaged for red fluorescent protein (RFP) using desynchronized laser sources for excitation and 600/65 nm filter for epi-reflected TPEF emission signals.

### Histology analysis of lung metastasis

Lung tissues were collected at 4 or 5 weeks after tumour implantation and kept in 10% buffered formalin. Paraffin-embedded sections of ~5 μm were mounted on glass slides and stained with hematoxylin and eosin. Histology samples were analyzed using an Eclipse E400 microscope (Nikon, Tokyo, Japan) and a Spot Insight Camera (Diagnostic Instrument, Sterling Heights, Michigan). Tumour colonies were identified based on the density of stained nuclei.

### Evaluating the impacts of VF conditioned medium on M109 cells

Visceral fat tissues (0.3 g) were collected from Balb/c mice at 8 to 12 weeks old, added to a culture dish containing 2 ml RPMI medium, and incubated at 37°C with 5% CO_2 _for 4 days. Conditioned medium (CM) was removed and added to approximately 1 million M109 cells in a 35 mm glass-bottom culture dish (MatTek, Ashland, MA, Cat. No. P35G-0-10-C). M109 cells in CM were kept in an incubator at 37°C with 5% CO_2_. The impacts of CM on M109 were monitored over time using CARS imaging and biochemical assays.

### Isolation of FFAs from CM or blood plasma

4 ml of hexane (Sigma-Aldrich, St Louis, MO, Cat. No. 296090) was added to 1 ml of CM or blood plasma and vortex-mixed. The mixture was centrifuge at 300 × g for 5 minutes to separate organic phase (top) from aqueous phase (bottom). Organic phase containing hexane and fatty acids was removed into a new container. Steady stream of nitrogen gas was used to evaporate hexane for 2 hours. FFAs were reconstituted into supplemented RPMI medium and used immediately or stored at -80°C. Aqueous phase which contains cytokines was used immediately or stored at -80°C.

### Probing fatty acids induced phase separation on cell membrane

To evaluate how different type of FFAs perturb tumour cell membrane, two membrane dyes were employed, DIOC18 (3,3'-dioctadecyloxacarbocyanine perchlorate, Invitrogen, Carlsbad, CA, Cat. No. D275) to probe liquid-ordered (L_o_) phase and Rh-DOPE (rhodamine B sulfonyl dioleoyl phosphatidyl ethanolamine, Avanti Polar Lipids, Alabaster, AL, Cat. No. 810150) to probe liquid-disordered (L_d_) phase on cell membrane. Final concentration of both DIOC18 and Rh-DOPE dyes used was at 25 μg/ml. For visualization of dye-labeled membrane, a 488 nm Argon laser was used to excite DIOC18 and a 543 nm Helium-Neon laser was used to excite Rh-DOPE. Emission signal was collected through a 520/40 nm filter for DIOC18 and 600/65 nm filter for Rh-DOPE. To avoid signal cross-over or possible FRET activity, the membrane was imaged sequentially using one laser source at a time. First, only the 488 nm Argon laser was used to visualize DIOC18 signal. Then, the Argon laser was blocked and the 543 nm Helium-Neon laser was used to visualize Rh-DOPE signal. The power of each laser was kept constantly at 80 μW at the sample.

### Three-dimensional M109 cells migration assay

In these assays, migration of M109 cells into explanted visceral fat tissues (VF) was examined. VF (0.3 g) was placed directly above the image area such that VF came into direct contact with M109 cells. After 24 hours of incubation, VF was imaged for invading M109 cells.

### Two-dimensional M109 cells migration assay

In these assays, membrane morphology and lipid content of migrating M109 cells along the bottom surface of a culture dish were examined. A polydimethylsiloxane (PDMS) well of 10 mm in diameter was used to seed ~10,000 M109 cells in 0.2 ml supplemented RPMI medium onto a culture dish for 24 hours. Seeded M109 cells were washed several times with fresh medium to remove unattached cells, then culture medium was removed with aspiration. The PDMS well holding seeded M109 cells was removed and the culture dish was filled with 2 ml of supplemented RPMI medium. VF (0.3 g) was placed in the culture dish away from the seeded M109 cells. M109 cells were allowed to migrate toward VF for 24 hours, then imaged with transmission and CARS microscopy.

### Extracellular matrix (ECM) M109 cell invasion assay

The ability of M109 cells to invade reconstituted basement membrane was evaluated using a QCM 96-Well Cell Invasion Assay kit (Chemicon, Temecula, CA, Cat. No. ECM 555). Experiments were performed according to manufacturer's protocol. We evaluated ECM invasion capability of four different pre-treated M109 cells toward four different chemoattractants. Pre-treated M109 cells are: 1) M109: M109 cells starved 24 hours by incubating in serum-free RPMI medium, 2) M109/CM: M109 cells in CM for 4 days, 3) M109/FFAs: M109 cells incubated for 4 days with extracted FFAs from CM, 4) M109/cytokines: M109 cells incubated for 4 days with extracted cytokines from CM. All pre-conditioned M109 cells were adjusted to 1 million cells per ml prior to ECM invasion assay. Chemoattractants are: 1) RPMI: complete RPMI medium with 10% supplemented fetal bovine serum, 2) CM: conditioned medium obtained by incubating 0.3 g VF in 2 ml of RPMI medium for 4 days, 3) FFAs: extracted FFAs from CM, 4) cytokines: extracted cytokines from CM. M109 cells were allowed to migrate across ECM membrane for 24 hours, then collected and assayed with CyQuant GR dye. Fluorescence reading was recorded with a multi-well fluorescence plate reader (Gemini XPS, Molecular Devices, Sunnyvale, CA) using a 480/520 nm filter set.

## Results and discussion

### Primary tumour growth was unaffected by a high fat diet

**Figure 1:**
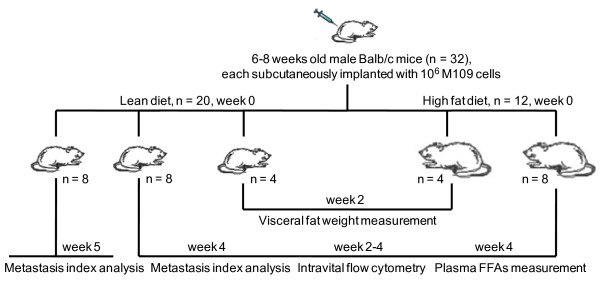
**A Balb/c mice model to evaluate the impact of excess lipid on cancer development**. Immediately after tumour implantation, mice were separated into two groups. One group was fed a lean diet (Harlan Teklad, Indianapolis, IN, Cat. No. 7001) with 4.25% fat and 3.82 Kcal/g, and the other group was fed high fat diet (Research Diets, New Brunswick, New Jersey, Cat. No. D12492) with 34.9% fat and 5.24 Kcal/g. Intravital flow cytometry and histology analysis were employed to enumerate circulating tumour cells (CTCs) in bloodstream and metastasized colonies in lung tissues, respectively. Additionally, primary tumour size, animal weight, weight of visceral adipose tissues, and concentration of plasma FFAs were also measured.

**Figure 2:**
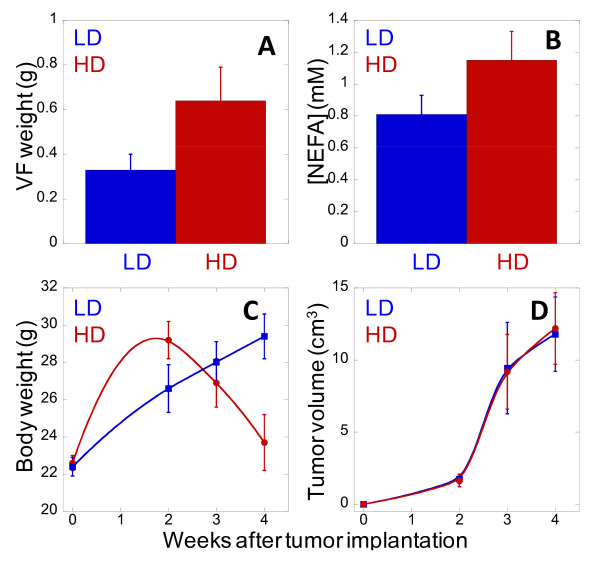
**Primary tumour growth in Balb/c mice is unaffected by high fat diet**. (**A**) Visceral adipose tissues (VF) weight as a function of lean (blue) and high fat (red) diets. VF tissues were extracted and weighted at 2 weeks after tumour implantation. (**B**) FFAs concentration as a function of lean (blue) and high fat (red) diets. Plasma was collected at 4 weeks after tumour implantation. (**C**) Body weight and (**D**) tumour volume as a function of time after tumour implantation. Error bars represent standard deviations across 8 mice (4 mice for Fig. 2A) measured in each diet group.

To evaluate the impact of excess lipid on cancer development, we employed a BALB/c mice cancer model where M109 lung cancer cells were implanted and grown subcutaneously into primary tumours (Fig. 1) [16]. Mice on lean diet (LD mice) were switched to a high fat diet (HD mice) immediately after tumour implantation. Tumour development in HD mice was monitored and compared with LD mice over time. Expectedly, HD mice exhibited higher visceral adiposity and plasma free fatty acids (FFAs) concentration than LD mice (Fig. 2A, B). HD mice also had more rapid weight gain than LD mice during the first two weeks on high fat diet (Fig. 2C). However, HD mice unexpectedly experienced severe weight decline from the third week; whereas, LD mice continued to gain weight until the fourth week (Fig. 2C). Surprisingly, the growth rate of primary tumours in HD and LD mice remained indistinguishable despite a striking difference in bodyweights (Fig. 2D). This observation suggested that severe weight loss in HD mice cannot be attributed to the growth rate of primary tumours.

### High fat diet induced early increase of circulating tumour cells

**Figure 3:**
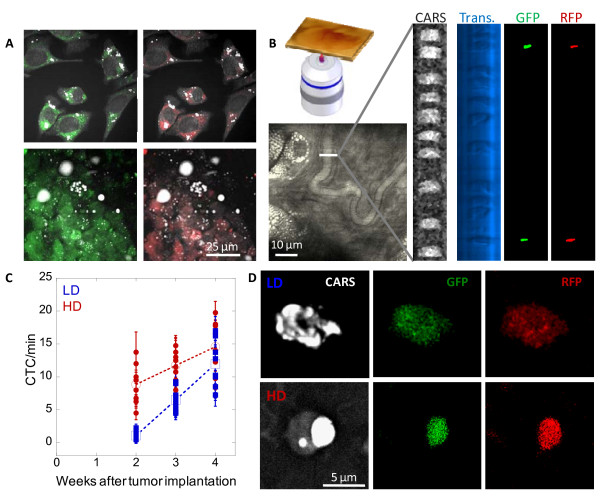
**High fat diet induces early increase of CTCs detectable with intravital flow cytometry**. (**A**) A dual-color M109 cell line which expresses both GFP and RFP. M109 cells in a tissue culture (upper panels) and in a primary tumour tissue (lower panels). (**B**) Intravital flow cytometry using a superficial vein of a mouse ear by dual-color confocal detection of CTCs. CARS: grey, transmission: light blue. CTCs were positively indentified based on both GFP (green) and RFP (red) signals generated by one-photon dual-laser excitations. Rightmost two panels were processed with ImageJ software to remove uncorrelated background noise. (**C**) High fat diet induced early increase of CTCs in the blood stream. Solid circles and squares represent average CTCs/minute measured in each HD and LD mouse, respectively. Error bars represent distribution of CTC/minute across four repeated measurements in each mouse. Open circles and squares represent average CTCs/minute of 8 HD and LD mice, respectively. No CTC detected on week 1 in mice of either diet group. (**D**) Isolated lipid-rich CTCs from LD (upper panels) and HD (lower panels) mice identified with CARS imaging for lipid and TPEF imaging for GFP (green) and RFP (red) expression.

Next, we examined how excess lipid due to high fat diet affects other stages of cancer development including cancer metastasis. To monitor the movement of cancer cells, we created a dual-labeled cancer cell line by stable transfection of M109 cells with a plasmid which expresses both green fluorescent protein (GFP) and red fluorescent protein (RFP) (Fig. 3A). This two-color labeling scheme enhanced tumour cell detection accuracy by minimizing false signal arising from autofluorescence intrinsic to many cell types. An early and critical stage in cancer metastasis is the escape of cancer cells into the bloodstream [17]. To enumerate circulating tumour cells (CTCs), we employed intravital flow cytometry, which is a proven robust method to monitor early stage of cancer metastasis [16]. Superficial microvessels in the ears of anesthetized mice were first identified using either CARS or transmission microscopy. Then CTCs were identified with both GFP and RFP signals using dual-laser confocal microscopy (Fig. 3B & supplemental video S1, see Additional file 1). We observed as much as 3-fold more CTCs in HD mice than LD mice at two weeks after tumour implantation (Fig. 3C). However, the difference in number of CTCs between two diet groups gradually declined and became indistinguishable by week 4 (Fig. 3C). Interestingly, CARS imaging revealed that all isolated CTCs from both diet groups had strong intracellular lipid accumulation (Fig. 3D). However, whereas lipid and cytoplasmic fluorescent proteins in all CTCs isolated from LD mice appeared evenly distributed throughout the cell, polar distribution was observed in all CTCs isolated from HD mice (Fig. 3D, Fig. S1 & Fig. S2, see Additional file 2). It is interesting to note that on average the diameter of a CTC was two-fold smaller than a M109 cell of primary tumours. Additionally, intracellular lipid appeared as a single droplet in CTCs rather than multiple droplets in M109 cells. Such differences could be attributed to mechanical stress exerted on CTCs during their flow through microvessels.

### High fat diet increased metastasis to lung tissues

**Figure 4:**
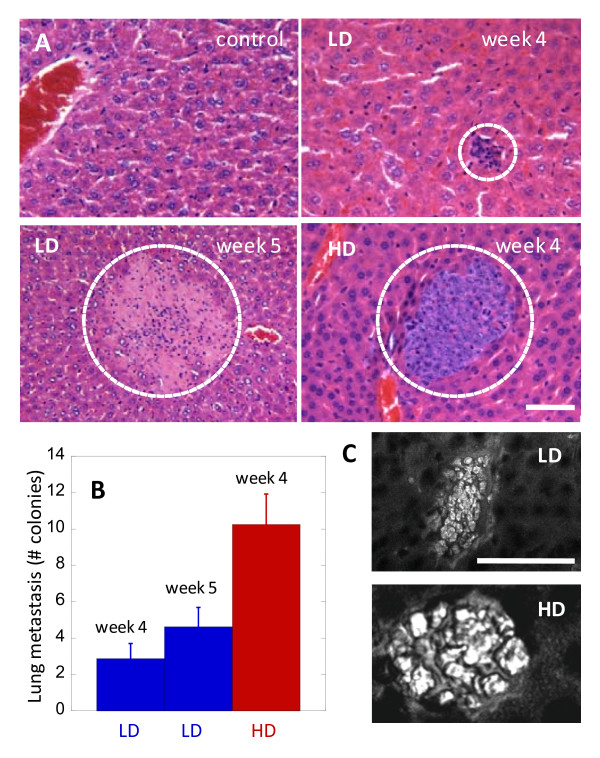
**High fat diet increases lung metastasis of lipid-rich cancer cells**. (**A**) Histology evaluation of lung tissues in a control mouse, LD mice at 4 and 5 weeks after tumour implantation, and a HD mouse at 4 weeks after tumour implantation. Dashed circles mark the locations of metastasized colonies. (**B**) Lung metastasis (number of metastasized colonies) as a function of diet. Error bars represent standard deviation across 8 mice analyzed. (**C**) Lipid-rich cancer cells in the lung tissues of LD (upper panel) and HD (lower panel) mice identified with CARS imaging (grey). Scale bars: 25 μm.

Another critical stage in cancer metastasis is the extravasation of CTCs to distant organs [17]. To assay for organ metastasis, we employed standard histology analysis of lung tissues and counted the number of metastasized colonies (Fig. 4A). We found as much as 4-fold more tumour colonies in lung tissues of HD mice than LD mice at 4 weeks after tumour implantation (Fig. 4B). To minimize the possibility of increased metastasis due to early CTC appearance, we also analyzed lung tissues from LD mice at week 5. While there was a slight increase in both the number and size of metastasized colonies in lung tissues, the overall lung metastasis of LD mice at week 5 remained at least 2-fold less than HD mice at week 4 (Fig. 4B). Notably, CARS imaging revealed significant lipid accumulation in all metastasized cancer cells of both LD and HD mice (Fig. 4C).

### FFAs induced membrane phase separation and intracellular lipid accumulation

**Figure 5:**
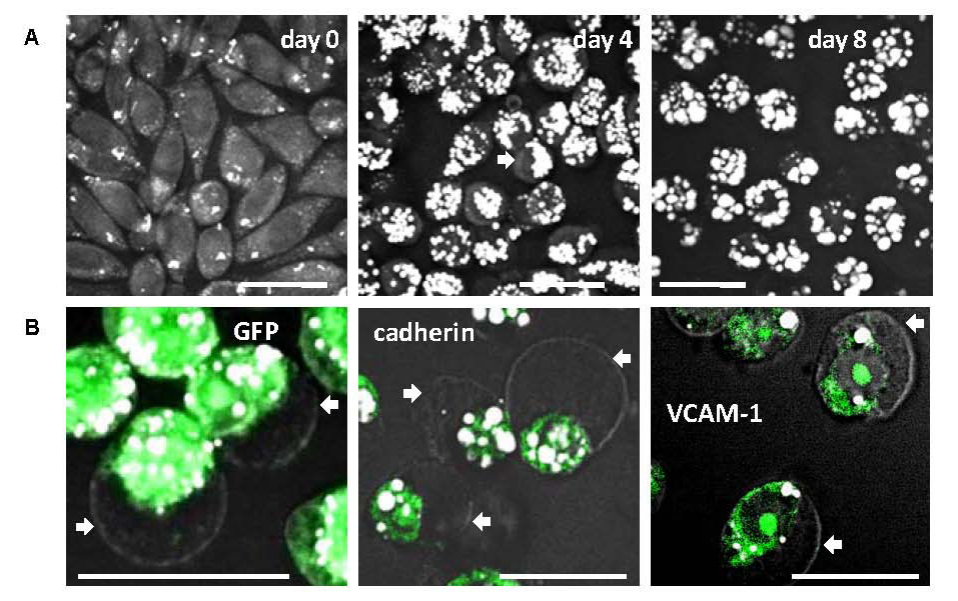
**VF conditioned medium induces intracellular lipid accumulation and cell polarity**. (**A**) CM induces lipid accumulation and polarity in lipid distribution in M109 cells. White arrows mark M109 cells with polarized lipid distribution. CM was obtained by incubating 0.3 g VF in RPMI medium for 4 days. (**B**) CM induces polarity in the distribution of cytoplasmic GFP (leftmost panel), and surface proteins cadherin (middle panel) and vascular endothelial adhesion molecule I, VCAM-1 (rightmost panel). Cadherin and VCAM-1 are visualized by immuno-labelled with FITC conjugated antibodies. Two M109 cell lines were used: one cell line was stably transfected with plasmid expressing GFP, and the other untransfected cell line was used to stain for cadherin and VCAM-1 CARS: grey; TPEF: green. Scale bars: 25 μm.

The presence of intracellular lipid in primary, circulating, and metastasized cancer cells raised the question on how excess lipid influences cancer cell behaviour leading to increased cancer metastasis. To address this question, we examined the effects of elevated plasma FFAs level and increased visceral adiposity on cancer cell cultures. Addition of plasma (data not shown) or conditioned media (CM) of explanted adipose tissues to M109 cells induced significant perturbations including accumulation of cytoplasmic lipid droplets, rounding of cell membrane, reduction of cell-cell contact, and polarity in the distribution of lipid droplets (Fig. 5A, B). Fluorescent imaging of labelled proteins of cytoplasm and plasma membrane further revealed an overall concentration of cellular content toward one cell pole (Fig. 5B & Fig. S3, see Additional file 2). Interestingly, polar distribution of cellular content in cultured cells strongly resembled CTCs isolated from HD mice (Fig. 3D).

**Figure 6:**
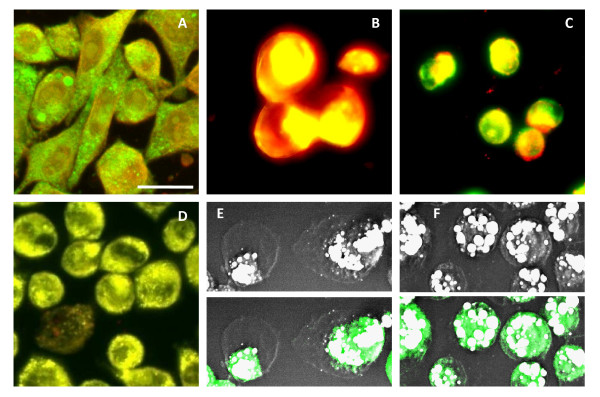
**Polyunsaturated and saturated FFAs induce membrane phase separation**. M109 cells were stained with membrane dyes DIOC18 (green) to probe liquid-ordered (L_o_) phase and Rh-DOPE (red) to probe liquid-disordered (L_d_) phase. (**A**) M109 cells exhibit uniform distribution of L_o _and L_d _phases. Yellow: overlaid of green and red signals. M109 cells pre-treated with (**B**) linoleic acids or (**C**) palmitic acids for 4 days exhibit membrane phase separation. (**D**) M109 cells pre-treated with oleic acids for 4 days exhibit uniform distribution of L_o _and L_d _phases. (**E**) CARS imaging can distinguish membrane phase separation induced by linoleic acids. Strong and weak CARS contrasts correspond to L_o _phase, where cytoplasmic GFP and other cellular content reside, and L_d _phase, where there is an absence of any cellular content, respectively. (**F**) CARS imaging cannot distinguish membrane phase separation induced by palmitic acids where cellular content, as marked by cytoplasmic GFP expression, is uniformly distributed. The final concentration of all FFAs used was kept at 50 μM to minimized lipotoxicity to M109 cells. Scale bars: 25 μm.

Using gas chromatography and mass spectrometry analysis, we identified four free fatty acids abundance in blood plasma and in CM as palmitic, stearic, oleic, and linoleic acids (Fig. S4, see Additional file 2). To examine the impact of each fatty acid on cancer cells, we separately added each fatty acid to M109 cell cultures. Using a pair of fluorescent membrane probes DIOC18 and Rh-DOPE to label liquid-ordered (L_o_) and disordered phases (L_d_) of the membrane [18], we observed polyunsaturated linoleic acid and saturated palmitic and stearic acid induced cell membrane separation into L_o _and L_d _phases (Fig. 6A–C). Cells with added polyunsaturated acid exhibited large cell shape with dominant L_d _phases (Fig. 6B). Conversely, cells with added saturated acid exhibited small cell shape with dominant L_o _phases (Fig. 6C). These observations were consistent with reported low and high membrane packing efficiency of polyunsaturated and saturated fatty acids, respectively [19]. On the other hand, no membrane phase separation was observed with oleic acid addition (Fig. 6D). This observation can be attributed to the ability of monounsaturated fatty acids to incorporate into both L_o _and L_d _phases [19]. In general, incorporation of excess fatty acids of any type into membrane perturbed membrane curvature, leading to the observed membrane rounding effect [18,19].

While all four free fatty acids induced intracellular lipid accumulation, only polyunsaturated linoleic acid induced cell polarity observable with CARS imaging (Fig. S5, see Additional file 2). Because cellular contents concentrated toward L_o _phase of the membrane, an increase in L_d _phase due to incorporation of polyunsaturated linoleic acid separated a single cell into two distinctive poles with one rich and the other poor in cellular contents. CARS imaging of content-rich cell poles which include cell membrane and lipid droplets and of content-poor cell poles which consist mainly of cell membrane yielded strong and weak CARS contrast, respectively (Fig. 6E). On the other hand, saturated FFAs-induced membrane separation was observable only with fluorescent imaging of phase probing dyes, but not with CARS imaging of lipid-rich structures (Fig. 6F). Hence, cell polarity observable with CARS imaging of CTCs isolated from HD mice strongly indicated incorporation of polyunsaturated FFAs into CTCs membranes (Fig. 3D). Furthermore, the presence of intracellular lipid in primary, circulating, and metastasized cells clearly indicated the exposure of cells to excess FFAs.

### FFAs induced cell polarity and increased surface adhesion capability

**Figure 7:**
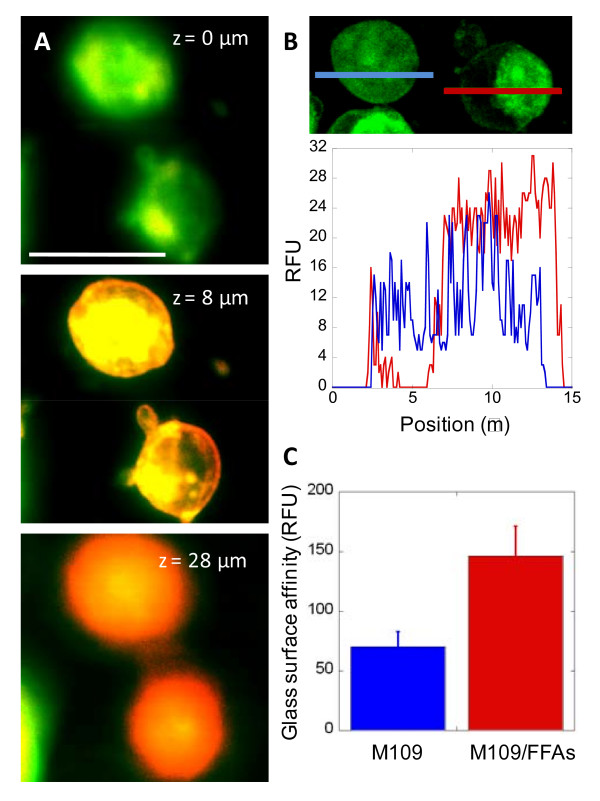
**Polarized cells induced by polyunsaturated FFAs exhibit increased surface affinity**. (**A**) Polarized cells exhibit preferred surface binding at L_o _phase of the membrane. M109 cells exposed to 50 μM linoleic acids for 4 days were first detached from culture dishes, then added back to a glass surface, stained with phase-probing dyes, and imaged. L_o _phase: green, L_d _phase: red, yellow: overlaid of green and red signals. Scale bars: 25 μm. Z = 0 um indicate glass surface cell membrane interface. (**B**) Surface protein density at L_o _phase of polarized cells is higher than average surface protein density of non-polarized cells. M109 cells exposed to 50 μM linoleic acids for 4 days were stained with FITC-conjugated antibodies to vascular cell adhesion molecules, VCAM-1. Random fluorescent unit (RFU), which represents the concentration of VCAM-1, is plotted against cell positions indicated by blue and red lines, which mark a non-polarized and a polarized cell, respectively. 3-D stacked image of 15 frames at 1 μm step size along vertical axis. (**C**) Polarized cells exhibit increased surface affinity. M109 cells untreated (blue) or treated with 50 μM linoleic acids for 4 days were first detached from the culture dishes, then added back to a glass surface for 45 minutes. Unattached cells were removed with gentle washing. Attached cells were lysed and assayed with CyQuant GR dye for nucleic acids. Fluorescent signals (RFU) are linearly correlated with cell number and indicate cell surface affinity.

Next, we investigated how polarity of CTCs in HD mice could contribute to the observed increase in lung metastasis by examining surface-binding capability of polarized M109 cells. M109 cells exposed to linoleic acids for four days were dissociated from culture dishes and added to glass-bottom dishes. We observed membrane surfaces at L_o _cell poles as the areas which adhered to glass surface during re-attachment (Fig. 7A). Because surface proteins concentrated into L_o _cell poles instead of over the entire cell surface, increases in the density of surface proteins in L_o _areas were observed (Fig. 7B & Fig. S6, see Additional file 2). Such increase in local density of surface protein could explain the preferential binding of L_o _cell poles to glass surfaces. We further compared surface-binding capability of polarity cells treated with linoleic acids and untreated cells with no polarity. We found approximately two-fold increase in glass-surface adhesion of polarized cells versus cells with no polarity (Fig. 7C). Our data are consistent with a previous report where treatment of linoleic acids increased adhesion of breast carcinoma cells to collagen-coated surface [20]. It is conceivable that polarized CTCs of HD mice could have stronger surface affinity than non-polarized CTCs of LD mice, which led to the increased CTCs extravasation and metastasis observed in HD mice (Fig. 4B).

### FFAs reduced cell-cell contact leading to increased chemotactic motility

**Figure 8:**
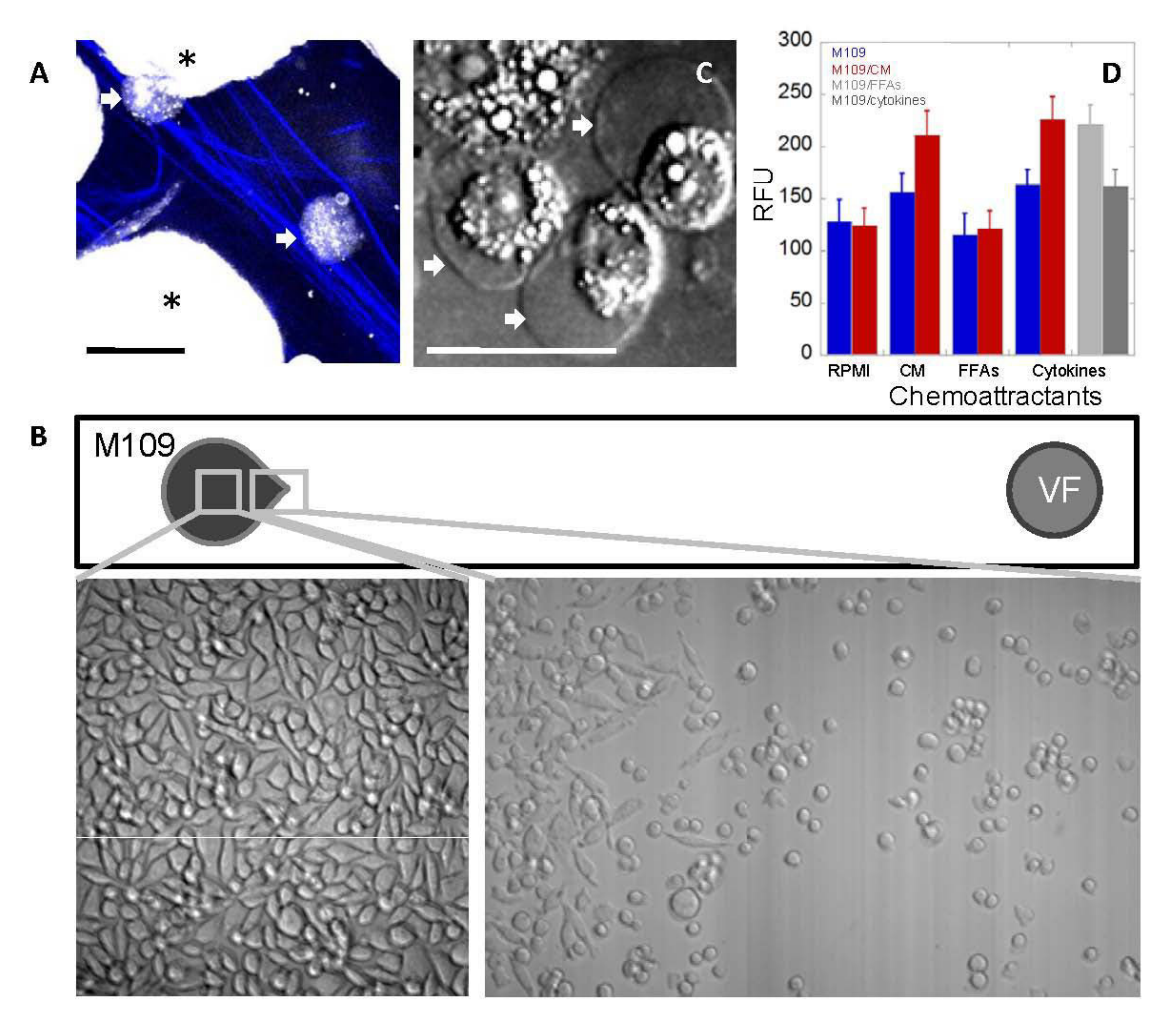
**Pre-treatment of M109 cells with FFAs increase ECM invasion capability**. (**A**) Lipid-rich M109 cells (arrows) associate with collagen type I fibres (blue) in a visceral adipose tissue (VF) after 24 hours of co-incubation. Asterisks (*) mark VF adipocytes. CARS: grey, SFG: blue. (**B**) Round cancer cells migrating toward a VF tissue along a 2-D bottom of a culture dish after 24 hours of co-incubation. Images taken with transmission microscopy. (**C**) CARS image of 2-D migrating cancer cells with intracellular lipid accumulation and cell polarity. Scale bars: 25 μm. (**D**) Pre-treatment of M109 cells with FFAs increases ECM invasion. ECM invasion assays for untreated M109 cells (blue) or M109 cells pre-treated with CM (red), pre-treated with only CM FFAs (light grey), or pre-treated with only CM cytokines (grey) moving toward chemoattractants: RPMI (supplemented growth medium), CM (VF conditioned medium), only CM FFAs, and only CM cytokines. RFU (relative fluorescent unit) is linearly correlated with cell number. Error bars represent standard deviation across 8 repeated assays.

Incorporation of FFAs into cell membrane had previously been shown to alter endothelial cell motility [21]. To examine the effects of FFAs on cancer cell motility, we performed several chemotaxis assays (Fig. S7, see Additional file 2). First, we incubated M109 cells with visceral adipose tissues and imaged for invading cells. By simultaneous CARS imaging of M109 cells and SFG imaging of collagen fibrils, we detected lipid-rich M109 cells at up to 25 μm from tissue surface after 24 hours of incubation (Fig. 8A). Secondly, we employed a two-dimensional migration assay to monitor cancer cells moving toward adipose tissues along the bottom of a culture dish. We observed migrating M109 cells within 24 hours of incubation (Fig. 8B). CARS imaging revealed migrating M109 cells with round morphology, high intracellular lipid accumulation, and cell polarity (Fig. 8C). Thirdly, we employed an extracellular matrix (ECM) invasion assay to evaluate the contribution of FFAs to M109 cell migration. M109 cells, untreated or pre-treated with CM, CM cytokines, or CM FFAs, were allowed to invade ECM membrane in the presence of growth medium, CM, CM cytokines, or CM FFAs (Fig. 8D). We found that CM FFAs by themselves did not serve as chemoattractants for M109 cells. Instead, M109 cells were attracted to CM cytokines (Fig. S8, see Additional file 2). However, M109 cells pre-treated with CM FFAs had significantly increased ECM invasion capability toward CM cytokines. Our chemotaxis assays suggested that altered M109 cell mobility was a consequence of direct effects of FFAs on cell membrane. It is conceivable that the reduction in cell-cell contact due to FFAs-induced membrane rounding rendered M109 cells more susceptible to chemotactic motility induced by CM cytokines (Fig. 8D).

Visceral adipose tissues are generally accepted as endocrine organs which regulate body lipid and energy homeostasis [22,23]. Increased visceral adipose mass is strongly associated with hyperlipidemia, elevated organ adiposity, altered free fatty acids metabolism, and abnormal adipokines secretion [23]. Here, we showed that secreted cytokines and FFAs from visceral adipose tissues exerted profound impacts on cancer cell motility and tissue invasion. Secreted cytokines attracted cancer cells; whereas, secreted FFAs perturbed cancer cell membrane leading to reduced cell-cell contact. Direct evidence of FFAs exposure was indicated by intracellular lipid accumulation in migrating cancer cells. While the negative impacts of increased visceral adipose tissues on cancer development had been focused mainly on altered chemical signaling induced by secreted cytokines, we showed that physical perturbations on cancer cell membrane induced by secreted FFAs also contributed to increased cancer aggressiveness. Taken together, our *in vitro *data supported a correlation between increased visceral adiposity or increased blood plasma FFAs level and increased cancer aggressiveness through the action of FFAs on cancer cells.

### FFAs impacts on M109 cells were observable in mammary cancer

**Figure 9:**
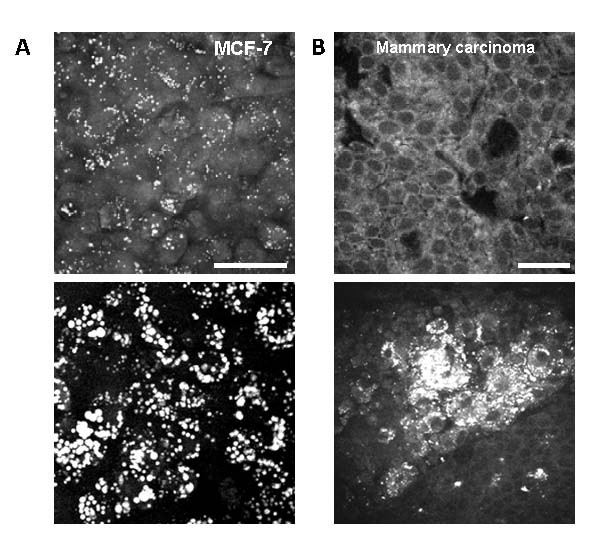
**CARS imaging reveals FFAs impacts on M109 cells are observable in mammary cancer**. (**A**) FFAs induced lipid accumulation and reduced cell-cell adhesion in human mammary cancer MCF-7 cell line. Upper panel: MCF-7 cells on day 0; lower panel: MCF-7 cells after 4 days of treatment with CM FFAs. (**B**) Lipid-rich cancer cells of a rat mammary carcinoma have disordered organization. Upper panel: lipid-poor rat mammary carcinoma; lower panel: lipid-rich rat mammary carcinoma. Positive CARS contrasts arise from lipid-rich membrane and intracellular lipid. Negative CARS contrasts indicate lipid-poor nuclei. Scale bars: 25 μm.

To determine whether the impacts exerted by FFAs remained extensible to other cancer cell lines, we examined the effects of FFAs on a human breast cancer cell line. We also examined lipid-rich breast tumour tissues of a rat cancer model. Using MCF-7 cell cultures, we observed addition of excess FFAs induced perturbations similar to those observed in M109 cell cultures including intracellular lipid accumulation and reduction of cell-cell contact (Fig. 9A). Using tissue biopsies of a Sprague-Dawley rat breast cancer model, where high fat diet and obesity were associated with increased carcinogenesis and aggressiveness of methylnitrosourea-induced breast cancer [15], we observed lipid-rich cancer cells exhibited disorganized formation in stark contrast to the highly ordered formation typical of lipid-poor cancer cells of epithelial origin (Fig. 9B). Such loss in tissue organization had been previously described as crucial for tissue invasion and metastasis by cancer cells [17]. Because FFAs induced lipid accumulation in cancer cells, lipid-rich cancer cells observed in rat breast cancer tissue biopsies could be a consequence of excess FFAs in tumour microenvironment. The comparable effects of FFAs on mammary cancer cells suggested the non-specificity of FFAs impact on cancer cell behaviours.

## Conclusion

In this study, we present a mechanistic dissection into the relationship between excess lipid and cancer aggressiveness. Using an animal cancer model, we observe mice with excess visceral adipose tissue or plasma FFAs due to a high fat diet experience early bodyweight loss, early appearance of high number of CTCs, and increased lung metastasis. CARS imaging reveals significant lipid accumulation in primary, circulating, and metastasized tumour cells. Furthermore, CTCs isolated from HD mice exhibit polarized distribution of lipid bodies and cytoplasmic proteins. Using M109 cell cultures, we show that FFAs induce intracellular lipid accumulation. FFAs incorporation into cell membrane causes membrane rounding, which leads to reduced cell-cell adhesion and increased tissue invasion. Moreover, polyunsaturated FFAs induce membrane phase separation and polarized distribution of surface, cytoplasmic, and cytoskeletal proteins. Cell polarity induced by polyunsaturated FFAs, and observed in CTCs of HD mice, increases surface binding capability. This observation could explain increased extravasation and metastasis of CTCs of HD mice. Taken together, both *in vitro *and *in vivo *studies strongly associate the effects of excess FFAs on cancer cell membrane with increased risk of cancer aggressive behaviours.

In addition to cancer cells, many other cell types also accumulate lipid in elevated serum FFAs condition [24]. Indeed, excess intracellular lipid accumulation in non-adipose tissues is associated with insulin resistance, cell death, and heart failure [25]. Currently, it is hypothesized that lipid accumulation is a general defence mechanism against lipotoxicity [24]. By channelling excess fatty acids toward lipid metabolism pathway and away from apoptosis pathway, cells improve their survival [24]. Many non-adipocyte cells, which have limited capacity to convert FFAs into neutral lipid, have been shown to alternatively incorporate FFAs into cell membrane, leading to increased cell motility [21], increased surface adhesion [20], and altered transmembrane signaling [26]. It is reasonable to believe that cancer cells also accumulate lipid to protect themselves against lipotoxicity (Fig. S9, see Additional file 2). However, unintended physical perturbations induced by FFAs on cancer cell membrane cause cancer cells to lose adhesion to neighbouring cells, become more susceptible to migration, and have increased opportunity to extravasate from bloodstream. Lipid-rich tumours could be a consequence of excess FFAs in tumour microenvironments. Therefore, treatment or classification of lipid-rich carcinoma should account for the risks induced by excess FFAs.

In our animal model studies, diet-induced increase of plasma FFAs level accelerates cancer metastasis. Nonetheless, the contribution of FFAs to cancer metastasis should be viewed in the context of a complex *in vivo *environment where multiple risk factors interact. For instance, our co-culture experiments show a strong influence of CM cytokines on cancer cell migration (Fig. 8D). Previous studies have also identified a number of other metastasis risk factors in tumour microenvironment including stem cells [27], fibroblasts [28], macrophages [29], collagen fibers [30], and others. Furthermore, FFAs have been shown to exert immunosuppressive effect by perturbing T cell membrane and inhibiting T cell signal transduction [26]. Given many cell types can be induced to accumulate intracellular lipid, it is unlikely that FFAs exert their impact specifically on cancer cells. To fully understand the impact of FFAs on cancer metastasis, the effects of FFAs on other cell types, particularly tumour stromal cells, should be comprehensively investigated.

Finally, the revelation of lipid-rich CTCs by CARS imaging could enable future development of label-free intravital CARS flow cytometry for early-stage diagnosis of cancer metastasis. Using our current nonlinear optical microscopy set-up, intravital CARS flow cytometry is yet able to discriminate lipid-rich CTCs from lipid-poor blood cells in a microvessel. Possible explanations could be attributed to CARS low sensitivity to flowing cells [31], interference of lipid-rich components of mouse skins [32] which descramble CARS signals arising from flowing cells, or un-optimized laser scanning speed per blood flow speed ratio [31]. However, recent advances in optics suggest that challenges for intravital CARS flow cytometry can be overcame. Such advances include adaptive optics which minimizes optical distortion in thick tissues [33], CARS signal generation from a single picosecond synchronously pumped optical parametric oscillator which increases penetration depth [34], and video-rate CARS microscopy which improves laser scanning speed [32]. Furthermore, advances in CARS endoscopy [35] also suggest possible clinical application of CARS imaging in the near future. The discovery of lipid-rich CTCs presented in this paper should facilitate translational development of CARS-based imaging tools for clinical cancer diagnosis.

## Abbreviations

CARS: coherent anti-Stokes Raman scattering; CTCs: circulating tumour cells; CM: conditioned medium of 0.3 g visceral fat tissue; ECM: extracellular matrix; FFAs: free fatty acids; GFP: green fluorescent protein; HD: high fat diet; LD: lean diet; RFP: red fluorescent protein; SFG: sum frequency generation; TPEF: two-photon excitation fluorescence; VF: visceral fat tissue.

## Competing interests

The authors declare that they have no competing interests.

## Authors' contributions

TTL and JXC designed experiments. TTL and TBH performed experiments and analyzed data. TTL and JXC wrote the paper. All authors read and approved final manuscript.

## Pre-publication history

The pre-publication history for this paper can be accessed here:

http://www.biomedcentral.com/1471-2407/9/42/prepub

## Supplementary Material

Additional file 1 **Intravital flow cytometry for the detection of circulating tumor cells.** A superficial blood micro-vessel of a Balb/c mouse on normal diet at 4 weeks after tumor implantation was selected for simultaneous confocal and transmission imaging. Movie acquired using xyt-scan mode at the speed of ~4 frames per second and a time window of 2 minutes. Green, GFP signal; red, RFP signal; blue, transmission signal. Movie represents raw, unprocessed data. Diameter of blood micro-vessel: 8 μm. Click here for file

Additional file 2 **Supporting figures S1–S9. **Additional supporting data Click here for file
