# Supplementary material for: Coherent anti-Stokes Raman scattering imaging of lipids in cancer metastasis
Source: BMC Cancer. 2009 Jan 30;9:42. doi: 10.1186/1471-2407-9-42 (PMC2640413; doi:10.1186/1471-2407-9-42)
Supplement: Additional file 2 — Supporting figures S1–S9. Additional supporting data [file 1471-2407-9-42-S2.pdf]

Supporting information

Coherent Anti-Stokes Raman Scattering Imaging of Lipids in Cancer Metastasis

Thuc T. Le,[1] Terry B. Huff,[2] Ji-Xin Cheng[1,2, 3§]

[1] Weldon School of Biomedical Engineering

[2] Department of Chemistry

[3] Purdue Cancer Center, Purdue University, West Lafayette, IN 47907, USA

[§]Corresponding author

Email addresses:

TTL: thuc@purdue.edu

TBH: tbhuff@purdue.edu

JC: jcheng@purdue.edu

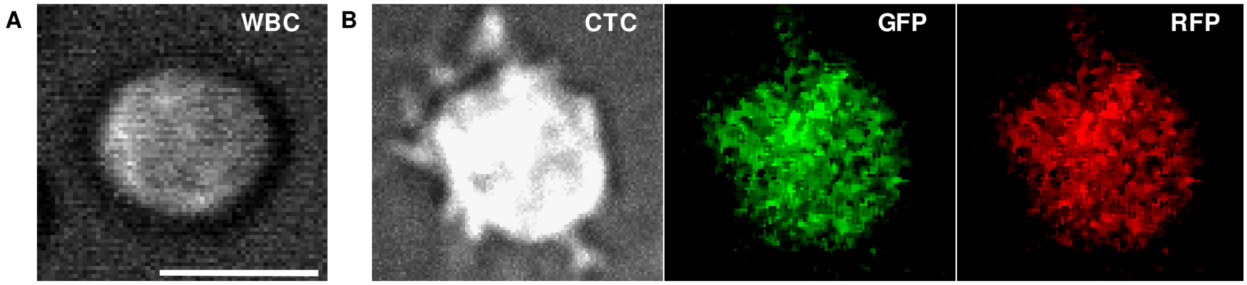

Figure S1. CARS imaging of a white blood cell and a circulating tumor cell. (**A**) A representative CARS image of a white blood cell (WBC). (**B**) A circulating tumor cell is identified using CARS imaging of lipid and TPEF imaging of GFP and RFP. In general, circulating tumor cells can easily be visualized due to strong CARS signal arising from high concentration of intracellular lipid. TPEF imaging of GFP and RFP fluorescent signals provides additional confirmation for CTC identification. Both WBC and CTC are isolated from a ND mouse. Scale bar: 5 µm.

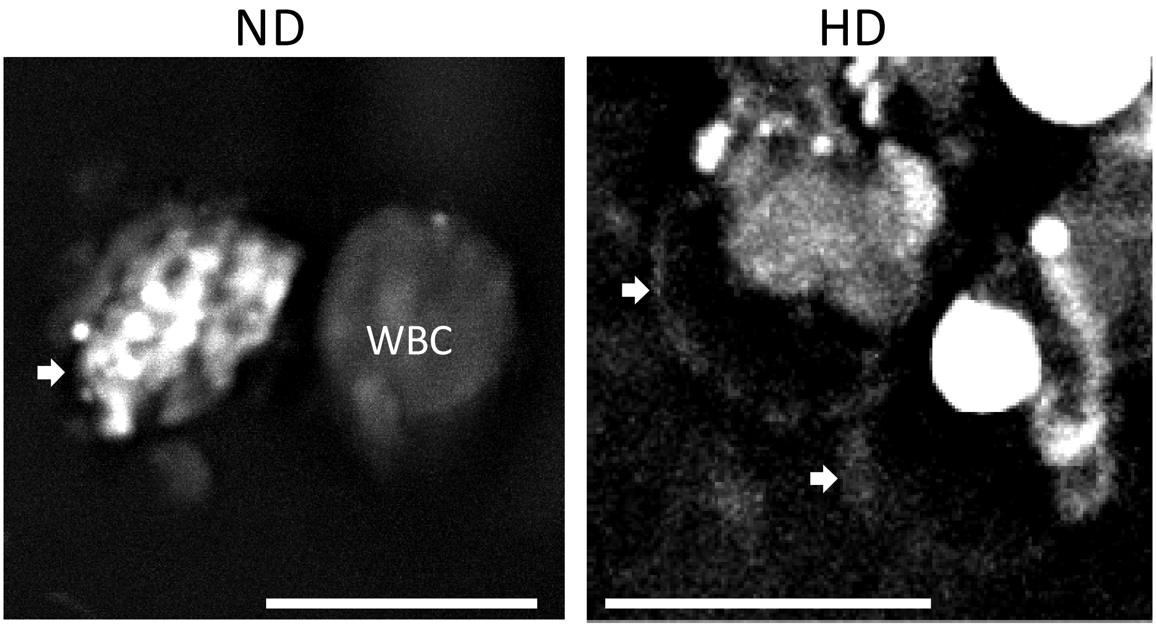

Figure S2. Membrane polarity of CTCs isolated from HD mice. (Left panel) A CTC (arrow) and a CTC isolated from a ND mouse. (Left lower panels) WBCs isolated from a ND mouse. (Right panel) Two polarized CTCs (arrows) isolated from a HD mouse. CTCs are distinguished from WBCs based on lipid signal as well as expression of GFP and RFP (not shown). Scale bars: 5 µm.

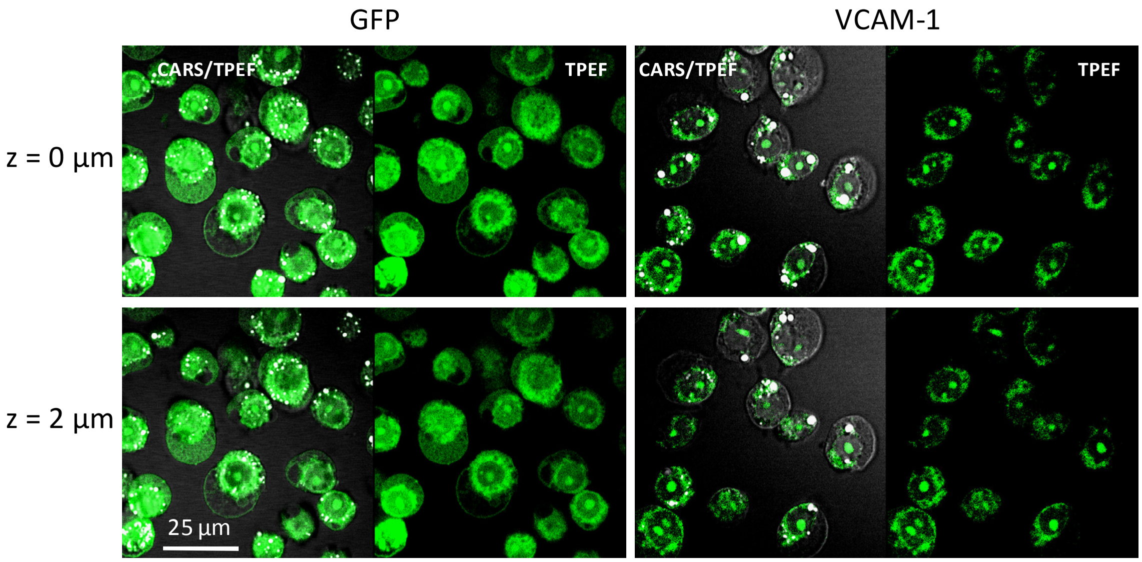

Figure S3. Depth imaging of cytoplasmic GFP and immuno-labeled labeled surface VCAM-1. (Left panels) One million M109 cells stably transfected with plasmid expressing GFP were incubated with 2 ml of conditioned media for 4 days, then imaged with CARS and TPEF. (Right panels) One million M109 cells were incubated with 2 ml of conditioned media for 4 days, then immuno-labeled for VCAM-1 and imaged with CARS and TPEF. CARS (grey), TPEF (green).

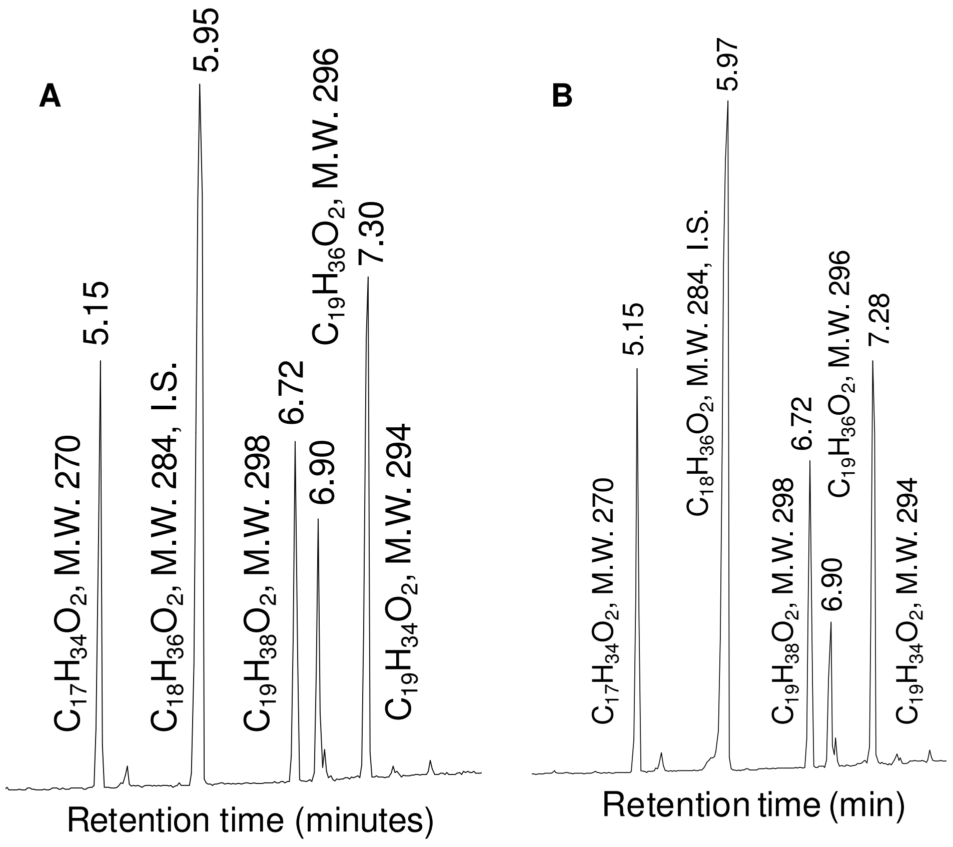

Figure S4. Gas chromatography and mass spectrometry analysis of free fatty acid methyl esters. (**A**) FFAs extracted from blood plasma of a LD mouse. (**B**) FFAs extracted from conditioned medium of visceral adipose tissues. Palmitic acid methyl ester: $C_{17}H_{34}O_2$, M.W. 270, R_t = 5.15 minutes (retention time). Heptadecanoic acid methyl ester (internal standard, I.S., Sigma-Aldrich, St Louis, MO, Cat. No. 51633): $C_{18}H_{36}O_2$, M.W. 284, R_t = 5.95 minutes. Stearic acid methyl ester: $C_{19}H_{38}O_2$, M.W. 298, R_t = 6.72 minutes. Oleic acid methyl ester: $C_{19}H_{36}$O2, M.W. 296, R_t = 6.90 minutes. Linoleic acid methyl ester: $C_{19}H_{34}O_2$, M.W. 294, 7.30 minutes. GC-MS was performed using a ThermoQuestGCQ mass spectrometer (ThermoFinnigan, San Jose, CA). Fatty acid methyl esters (FAME) were separated using a capillary column DBWAX (J&W Scientific, Folsom, CA). Electron energy: 70eV and ion source temp: 200°C. Chemical ionization gas: isobutane. Injector temperature: 250°C. In addition, GC-MS analysis of a standard fatty acid methyl ester (FAME) mixture (Supelco, Bellefonte, PA, Cat. No. 18919-1AMP) was performed to serve as a standard reference (data not shown).

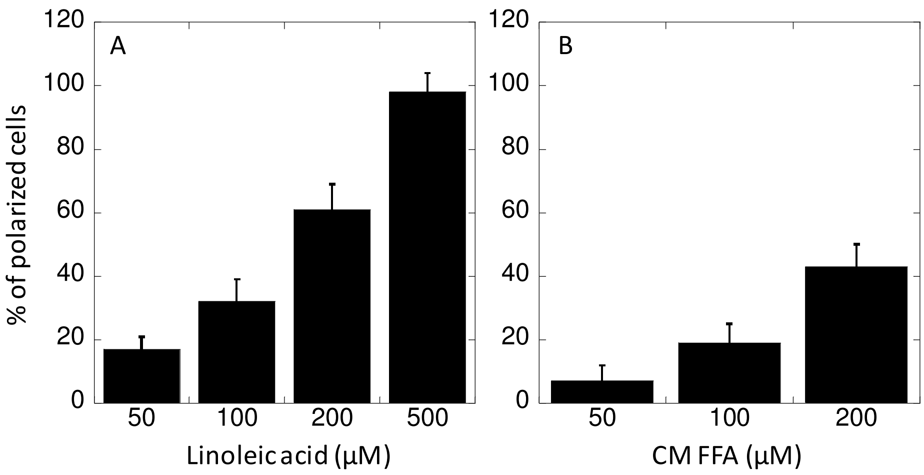

Figure S5. Percentage of polarized cells as a function of fatty acid concentration. (**A**) Percentage of polarized M109 cells as a function of linoleic acid concentration. (**B**) Percentage of polarized M109 cells as a function of free fatty acids concentration in conditioned media. 1 million M109 cells were incubated with 2 ml of media containing either linoleic acid or CM FFA for 4 days. The fractions of cells with polarized membrane are identified using CARS imaging. Error bars represent distribution across 3 repeated experiments.

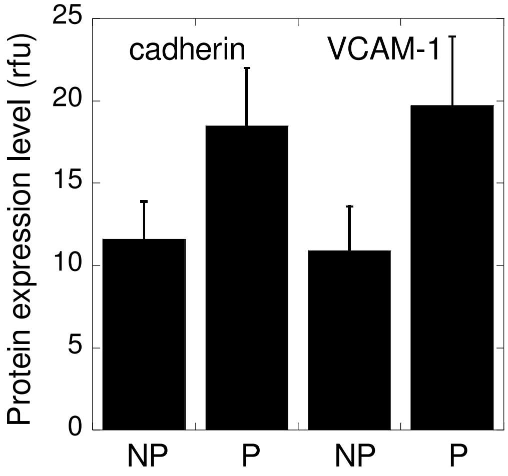

Figure S6. Surface protein expression level in non-polarized (NP) and polarized (P) M109 cells. Cadherin and VCAM-1 expression levels are measured by average pixel intensity of (a) the entire cell surface area for non-polarized cells, or of (b) liquid-ordered poles for polarized cells. M109 cells were incubated with 50 µM of linoleic acid for 4 days, then assayed for cadherin and VCAM-1 expression by immuno-labeling and TPEF imaging. Depth imaging at 1 µm step size along vertical axis were performed to visualize the entire 3-D cell surface. Average pixel intensity was obtained using Fluoview image processing software. Error bars represent distribution across one hundred cells assayed. RFU stands for random fluorescent units.

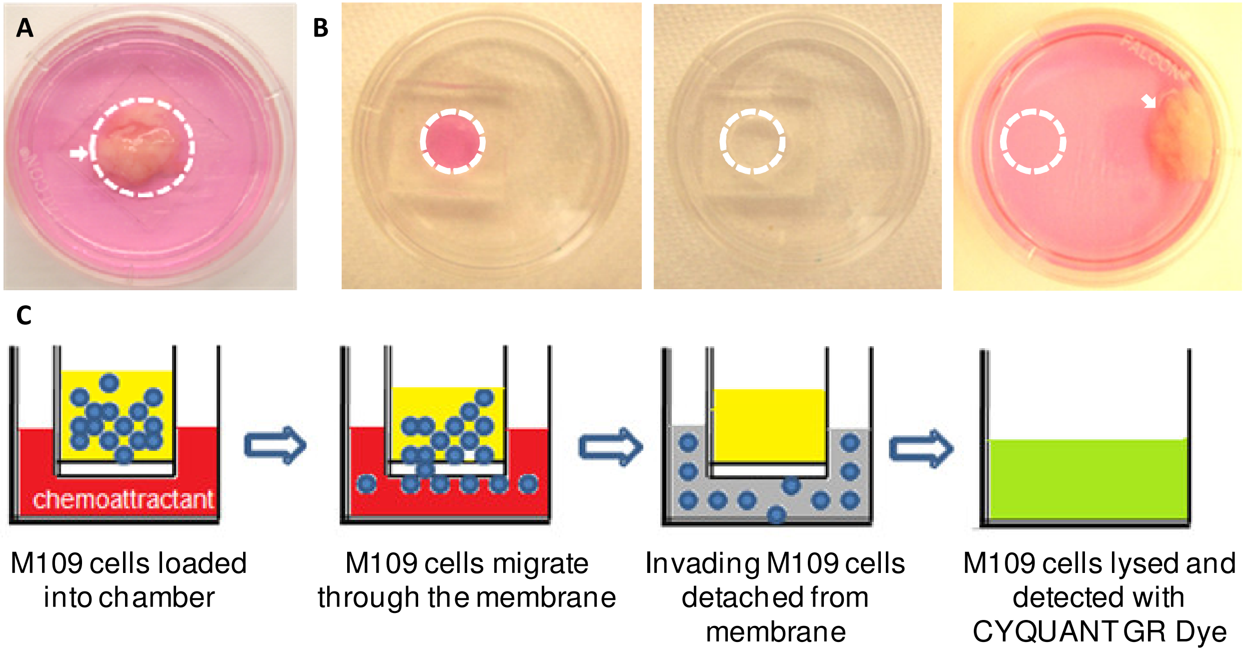

Figure S7. Experimental set-ups for chemotactic assays. (**A**) Three-dimensional migration assay. VF (0.3 g, arrow)) was placed directly above the image area (dashed circle) such that VF came into direct contact with M109 cells. After 24 hours of incubation, visceral fat tissue was imaged for invading M109 cells. (**B)** Two-dimensional migration assay. A polydimethylsiloxane (PDMS) well of 10 mm in diameter was used to seed ~10,000 M109 cells in 0.2 ml RPMI medium for 24 hours (leftmost panel). Unattached M109 cells were removed with several washes (middle panel). PDMS well was removed and 2 ml of RPMI medium was added. VF (0.3 g, arrow) was added to the far end of the culture dish (rightmost panel). M109 cells were allowed to migrate toward visceral fat tissue for 24 hrs, then visualized with transmission or CARS microscopy. Dashed circle indicates the original seeded area for M109 cells. (**C**) Extracellular matrix (ECM) invasion assay. A QCM 96-well cell invasion assay kit is employed (Chemicon, Temecula, CA, Cat. No. ECM 555). Experiments were performed according to manufacturer's protocol.

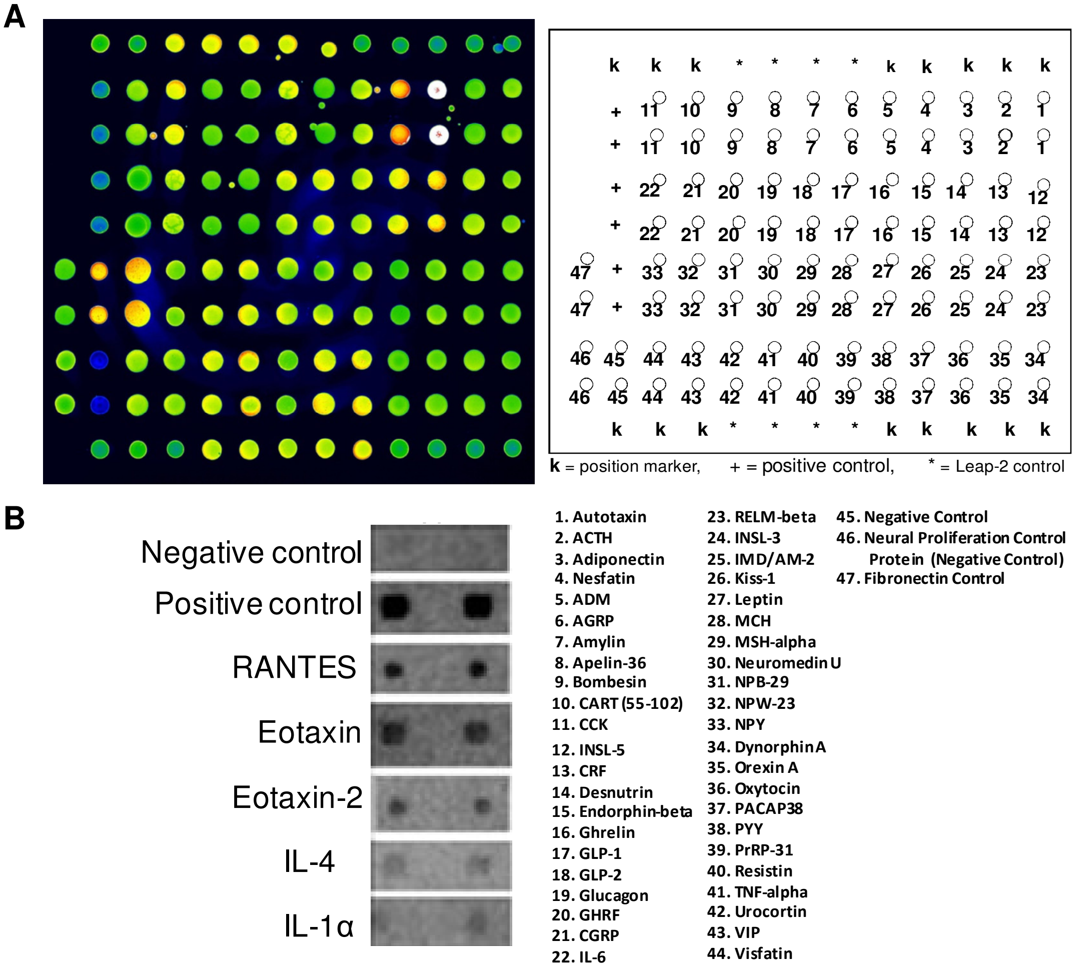

Figure S8. Identification of cytokines in VF conditioned medium. (**A**) A peptide biomarker array (Phoenix Pharmaceuticals, Belmont, CA, Cat. No. OC-K-003F) identifies the presence of a number of adipokines including: adiponectin, TNF-α, desnutrin, leptin, resistin, vistafin, ghrelin, INSL-3, IL-6, and others. (**B**) A cytokine antibodies array (RayBiotech, Norcross, GA, Cat. No. AAM-CYT-3-4) identifies the presence of chemoattractant and inflammation peptides including RANTES, eotaxin, eotaxin-2, IL-4, and IL-1α. Experiments were performed according to manufacturers' protocols.

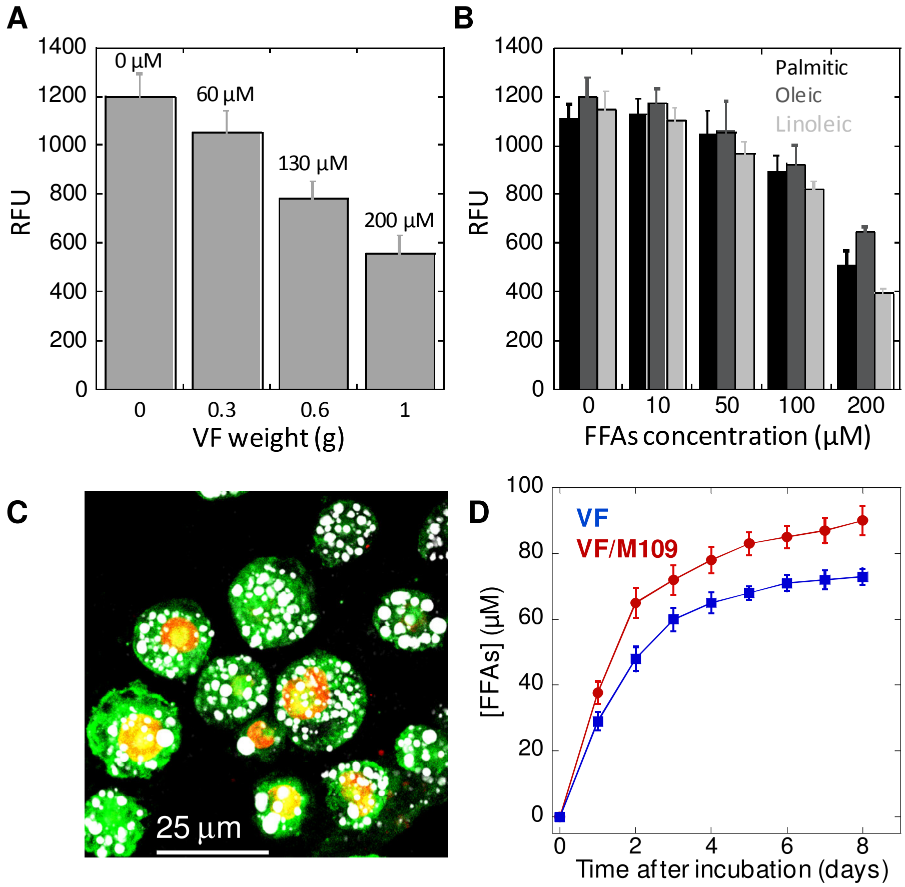

Figure S9. Excess FFAs induce lipotoxicity in M109 cells. (**A**) M109 cell number as a function of VF weight. Conditioned media (CM) after 4 days of incubation with of 0.3 g, 0.6 g, and 1 g of VF were added to culture dishes containing approximately 1 million M109 cells each. M109 cell number was quantified at 4 days after CM addition. The measured concentrations of FFAs in CM are indicated above the columns. (**B**) M109 cell number as a function of individual FFA after 4 days of incubation. Cell growth was quantified using a quick cell proliferation assay kit according to manufacturer's protocol (BioVision, Mountain View, CA, Cat. No. K301-500). RFU stands for random fluorescent unit and is directly proportional to the number of cells. Error bars represent distribution across 4 repeated experiments. (**C**) M109 cells undergo apoptosis after 4 days of incubation with CM of 1g VF or approximately 200 μM FFAs. Apoptosis assay was performed using an apoptosis kit according to manufacturer's protocol (Invitrogen, Carlsbad, CA, Cat. No. V13241). Grey: F-CARS signal. Red: propidium iodide TPEF signal. Green: AlexaFluor488-annexin V TPEF signal. From these data, CM of 0.3 g VF was chosen for all experiments to minimize lipotoxicity to M109 cells. (**D**) FFAs secretion from 0.3 g VF into CM as a function of days after incubation. VF (blue): 0.3 g VF in RPMI medium. VF/M109: 0.3 g VF in a culture dish containing 1 million M109 cells. Error bars represent distribution across 4 repeated experiments.
